# Assessing the Anti-Aging and Wound Healing Capabilities of *Etlingera elatior* Inflorescence Extract: A Comparison of Three Inflorescence Color Varieties

**DOI:** 10.3390/molecules28217370

**Published:** 2023-10-31

**Authors:** Chutima Sinsuebpol, Titpawan Nakpheng, Teerapol Srichana, Somchai Sawatdee, Weerachai Pipatrattanaseree, Kanokporn Burapapadh, Narumon Changsan

**Affiliations:** 1 College of Pharmacy, Rangsit University, Pathum Thani 12000, Thailand; chutima.si@rsu.ac.th (C.S.);; 2 Drug Delivery System Excellence Center, Department of Pharmaceutical Technology, Faculty of Pharmaceutical Sciences, Prince of Songkla University, Songkhla 90112, Thailandteerapol.s@psu.ac.th (T.S.); 3 Drug and Cosmetics Excellence Center, Walailak University, Thasala, Nakhon Si Thammarat 80160, Thailand; somchai086@hotmail.com; 4 Regional Medical Sciences Center 12 Songkhla, Ministry of Public Health, Songkhla 90000, Thailand; weerachai.dmsc@gmail.com

**Keywords:** torch ginger, *Etlingera elatior*, anti-aging, wound healing, inflorescence color

## Abstract

Torch ginger, *Etlingera elatior*, is a Zingiberaceae plant with various red, pink, and white inflorescence. The wound healing potential and anti-aging effects of freeze-dried torch ginger inflorescence extracts (FTIEs) from three varieties were compared. The red FTIE had the highest content of phenolic, flavonoid, caffeoylquinic acid, and chlorogenic acid, followed by the white and pink FTIE. Consistent with the chemical constituents, the red FTIE demonstrated the greatest capacities for free radical scavenging, anti-tyrosinase, and anti-collagenase activity, followed by the white and pink FTIE. In cell-based studies, FTIEs displayed cytotoxicity to B16F10 melanoma cells, with the red FTIE showing the greatest activity (LC_50_ of 115.5 μg/mL). In contrast, the pink and the white FTIEs had less cytotoxicity impact. Nonetheless, at 1000 μg/mL, all three FTIE variants were safe on L929 fibroblasts or RAW 264.7 monocyte cells. White FTIE (500 μg/mL) exhibited the highest activity in stimulating collagen production and the greatest impact on cell migration, whereas the pink and red FTIE had a lesser effect. All FTIEs slightly suppressed the pro-inflammatory cytokines produced by lipopolysaccharide-stimulated monocytes, with no significant variation between FTIE variants. In conclusion, all FTIEs revealed promising potential for anti-aging cosmeceuticals and wound care products at specific concentrations.

## 1. Introduction

As individuals age, there is a notable alteration in the functioning of human organs. Skin aging is a natural process that can be recognized more easily than changes in any other organ. As a result of intrinsic factors such as hormonal changes, cellular senescence, and genetics, the skin naturally ages and becomes saggy and wrinkled. In addition to the naturally occurring aging processes, skin aging is also significantly influenced by photoaging, which is triggered by environmental factors such as exposure to UV light, pollution, and cigarette smoke. The aging processes, including both natural and photo-aging, result in an elevation of reactive oxygen species (ROS) levels, leading to oxidative stress within the skin. This oxidative stress subsequently triggers the synthesis of matrix metalloproteinases (MMPs) [1,2]. MMPs play a significant role in the degradation of the extracellular matrix (ECM) found in the dermis, specifically targeting components such as collagen and elastin [1,2]. Collagenase, namely MMP 1, is classified as a member of the MMP family and is known for its ability to destroy collagen enzymatically. Furthermore, oxidative stress can also lead to a decrease in collagen synthesis [1,2]. All these factors contribute to an increase in collagen degradation and a reduction in collagen synthesis. Hence, aging leads to several alterations in the physical, morphological, and physiological characteristics of the skin which are commonly observed in adult individuals. These alterations include the development of wrinkles, a decline in skin elasticity, uneven pigmentation and discoloration, dryness, and a slower rate of regeneration and healing [1,2]. Therefore, employing compounds exhibiting antioxidant activity, anti-collagenase activity, and the capacity to stimulate collagen formation represents the potential for development as a cosmeceutical product for mitigating skin aging. In addition, a substance that can stimulate collagen formation could accelerate the process of wound healing and thus serve as an advantageous component in wound healing treatment.

Torch ginger, or *Etlingera elatior* (Jack) R. M. Smith, is a plant in the Zingiberaceae family. It is widely planted throughout Southeast Asia and is native to Thailand, Indonesia, and Malaysia, where it is known as “*kaalaa*”, “*kecombrang*”, and “*bunga kantan*”, respectively [3,4]. It is an edible plant in which its flowers and leaves are frequently used as spices to flavor food or are eaten raw for their therapeutic benefits. Furthermore, it is traditionally used to treat earaches and clean wounds as an ingredient in local products such as soap, shampoo, and perfume [3,5]. Large and beautiful inflorescences of torch ginger have conspicuous bracts of the principal three colors of red, pink, and white [6,7]. The variation in flower color is mostly due to the varied combinations of three major pigment groups: flavonoid, carotenoid, and betalanin. Various flavonoid derivatives range from colorless molecules like flavonols to colored pigments like anthocyanins, tannins, and proanthocyanidins. Flavonoids, in addition to coloring flowers and fruits, offer a variety of biological activities, including antioxidant, anticancer, and anti-swelling effects [8]. Furthermore, there are several reports on secondary metabolites from torch ginger, such as phenols, flavonoids, glycosides, saponins, tannins, steroids, and terpenoids, which demonstrated several bioactivities such as anti-hyperglycemic, anti-hyperuricemic, anti-inflammatory, anti-microbial, antioxidant, tyrosinase inhibition, anti-tumor activity, and hepatoprotective activity [4,5,7,9]. Whangsomnuek et al. [10] also demonstrated the skin advantages of torch ginger flower extract, indicating that it could be a natural source of anti-aging and anti-wrinkle active components in cosmetic products.

According to our knowledge, there is no published report on the distinct bioactivities and phytochemical compositions of the three flowering varieties of torch ginger. Consequently, this study aimed to establish a potential relationship between the flower color variety of torch ginger and its potential applications for use as a cosmeceutical active ingredient or in wound healing preparations.

## 2. Results and Discussions

**Figure 1:**
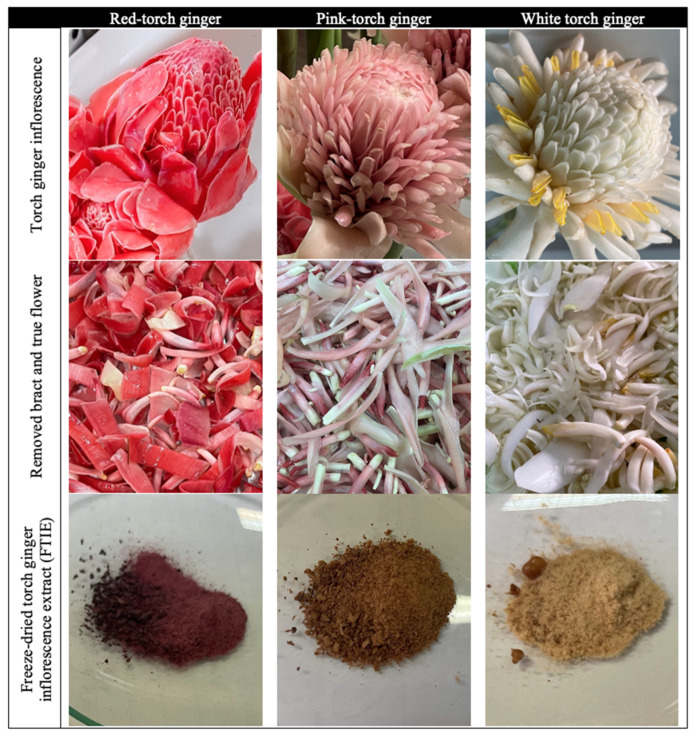
Inflorescence, removed bract and freeze-dried torch ginger inflorescence extract (FTIE) powder of the red, pink, and white torch ginger variety.

The white-, pink-, and red-inflorescence varieties of torch ginger were successfully extracted using 50% methanol, and the extraction was then freeze-dried to produce a dry powder. The obtained powder was free-flowing and resembled the actual fresh inflorescence color, as shown in Figure 1.

The chemical composition, free radical scavenging, anti-tyrosinase and anti-collagenase enzyme activity, cell viability, fibroblast cell-based collagen production and migration, and monocyte cell-based anti-inflammatory activity of freeze-dried torch ginger inflorescence extracts (FTIEs) were compared across three color variants.

### 2.1. Total Phenolic, Flavonoid, Caffeoylquinic Acid, Chlorogenic, and Rutin Content of FTIEs

Different flower colors confer different pigments, and several flavonoid derivatives participate in this pigment biosynthesis pathway, e.g., from colorless compounds such as flavonols to colored pigments such as anthocyanins, tannins, and pro-anthocyanidins. However, flavonoid compounds also display various biological activities, including antioxidant, anticancer, and anti-swelling [8]. As a result, different flower colors may offer distinct biological properties. Caffeoylquinic acids, or CQAs, are secondary metabolites found in several plants used as food and medicine. CQA exists in three isomer forms. The most common one is chlorogenic acid (CGA or 5-CQA). CGA is a natural antioxidant used in food, medicine, and cosmetics. According to Chan et al. [11], the torch ginger leaf is abundant in total phenolic content (TPC) and CQA content. Rutin, or rutoside, or quercetin-3-rutinoside is a widely distributed flavonol in plants. It has been demonstrated to have various pharmacological properties, including antioxidant, cytoprotective, and wound-healing actions [12].

In this study, the total phenolic, flavonoid and caffeoylquinic acid content was determined using a UV-Vis spectrophotometer, and the absorbance at the specified wavelength was calculated to be equivalent to standard gallic acid (gallic acid equivalent, GAE), rutin (rutin equivalent, RE), and chlorogenic acid (chlorogenic acid equivalent, CGAE) in the unit mg equivalent/g FTIE, as shown in Table 1.

**Figure 2:**
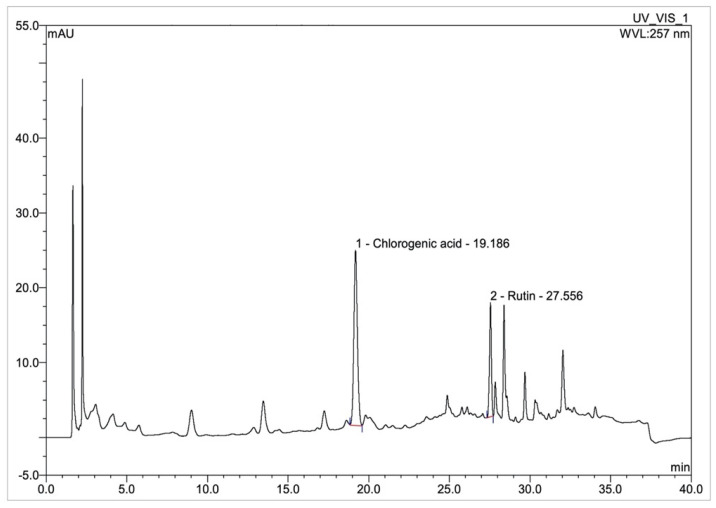
HPLC chromatogram of the red-FTIE, in which CGA and rutin were detected at 19.19 and 27.56 min.

Chlorogenic acid (CGA) and rutin content in FTIEs were analyzed using high-performance liquid chromatography (HPLC), as displayed in Figure 2. As depicted in the chromatogram, CGA and rutin were detected at 19.19 and 27.56 min (retention time), respectively, along with several unidentified chemical compounds.

The results revealed that FTIE, in all variants, demonstrated a high content of phenolic, flavonoid, caffeoylquinic acid, CGA, and rutin. The red-FTIE had significantly higher levels of phenolic, flavonoid, caffeoylquinic acid, and CGA than the white-FTIE, while the pink-FTIE showed the lowest levels. In contrast, rutin content was most prevalent in the pink-FTIE, followed by the red-FTIE and the white-FTIE. In addition, the HPLC chromatogram (Figure 2) revealed several peaks of unidentified chemical compounds, indicating that FTIEs are composed of various chemical compounds.

### 2.2. Cell Free-Antioxidant, Anti-Tyrosinase and Anti-Collagenase Activity of FTIEs

Aging processes cause an increase in reactive oxygen species (ROS) and oxidative stress in the skin, resulting in physical, morphological, and physiological skin changes associated with mature skin, such as wrinkles, a loss of elasticity, uneven pigmentation, and discoloration, dryness, slower regeneration, and healing [13]. Therefore, substances that have the potential to inhibit free radicals will delay the aging process and a variety of skin-layer changes.

The ability of FTIEs to scavenge free radicals was investigated using the DPPH and ABTS methods. The results of antioxidant activity tests, as the concentration required to achieve 50% free radical inhibition activity (IC_50_), measured in μg/mL, conducted on three variants of FTIE, are presented in Table 2. All three FTIE variants showed significant antioxidant activity, with IC_50_ values below 105 μg/mL for the DPPH system and below 500 μg/mL for the ABTS system. In both the DPPH and ABTS systems, the red-FTIE showed the highest antioxidant activity, while the pink-FTIE had the lowest. These results were in accordance with the total phenolic, flavonoid, CQA, and CGA content findings. CGA, a natural antioxidant, exhibited strong DPPH free radical scavenging abilities comparable to those of standard gallic acid. Rutin was found to exhibit powerful antioxidant activity, although was comparatively weaker than that of CGA. Even though it was discovered that the pink-FTIE had the largest amount of rutin, it did not exhibit excellent antioxidant activity when compared to the other two variants due to the low ratio of rutin to other chemicals and the moderate antioxidant activity of rutin.

Tyrosinase is an enzyme that contributes to the synthesis of melanin, the skin pigment responsible for skin color. The tyrosinase enzyme has been accepted as the rate-limiting step of the melanin production pathway. Therefore, tyrosinase enzyme inhibition is the target of melanin synthesis inhibition. All variants of FTIE had low activity against the tyrosinase enzyme, as the IC_50_ values were higher than 3000 μg/mL. There was no difference between the IC_50_ values of the red and the white FTIE (*p*-value > 0.05). Similar to the findings for antioxidant activity, the pink FTIE had the lowest anti-tyrosinase activity. Kojic acid is utilized as a positive control, with an IC_50_ of 53.60 ± 11.28 μg/mL. However, it was shown that CGA and rutin had no inhibitory effect on tyrosinase activity within the studied concentration range (less than 1000 μg/mL). The same results were seen in the cell-free anti-collagenase test as with the other activities. The red FTIE showed the most activity inhibiting the collagenase enzyme (IC_50_ of 255.68 ± 19.06 μg/mL), which was more effective than the control group of EGCG (IC_50_ of 385.40 ± 25.54 μg/mL). CGA displayed weak activity against collagenase enzyme inhibition. However, the pink FTIE and rutin did not demonstrate any detectable anti-collagenase activity within the range of examined concentrations. These results demonstrated that CGA and rutin are not the primary compounds responsible for the anti-collagenase activity of FTIEs.

In conclusion, cell-free studies that focus on the anti-aging cosmeceutical activity of FTIE, including antioxidant activity, anti-tyrosinase activity, and anti-collagenase activity, revealed that the red-FTIE exhibited more promising activity than the white- and the pink-FTIE. Due to its high-phenolic, flavonoid, caffeoylquinic acid, and chlorogenic content, the red FTIE demonstrated the maximum activity levels for all categories. The findings correlate with previous research conducted by Chaikhong et al. [14] on the potential anti-aging properties of Thai plants. The study revealed that compounds possessing certain amounts of total phenolic content and exhibiting antioxidant activity can contribute to the inhibition of tyrosinase and elastase enzymes, thereby providing additional support for anti-aging mechanisms. 

### 2.3. Cell-Based Assays

#### 2.3.1. Cell Viability Assay

**Figure 3:**
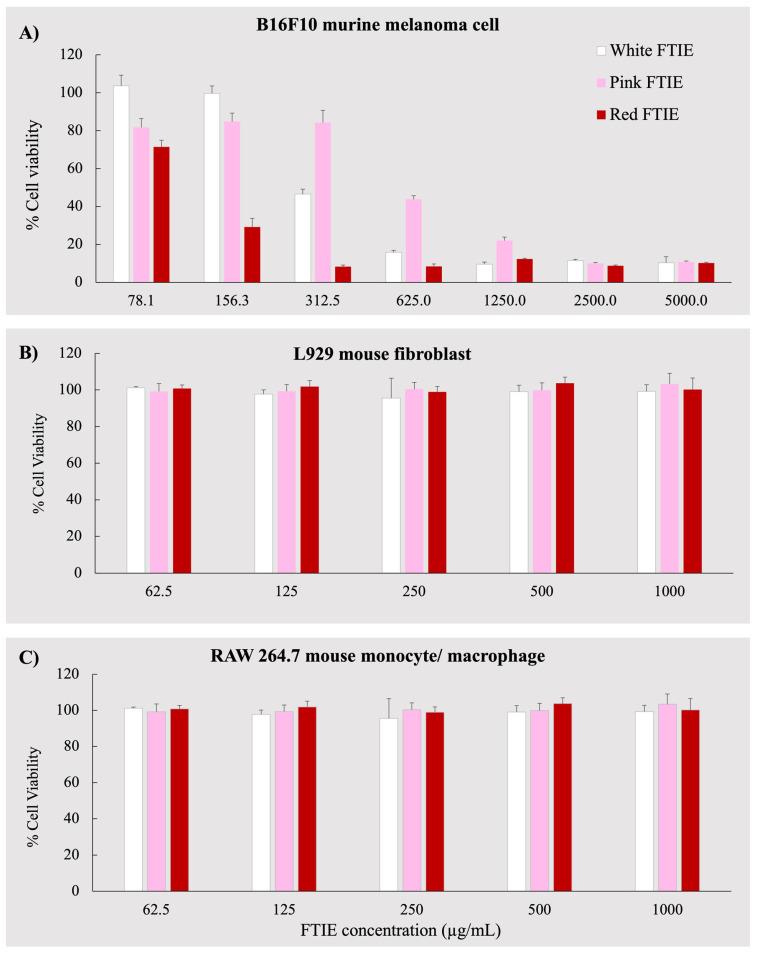
Percentage of cell viability of (**A**) B16F10 murine melanoma cell, (**B**) L929 mouse fibroblast cell, and (**C**) RAW264.7 mouse monocyte/macrophage cell upon exposure to FTIEs at various concentrations. Data expressed as a mean ± SD, *n* = 4.

**Figure 4:**
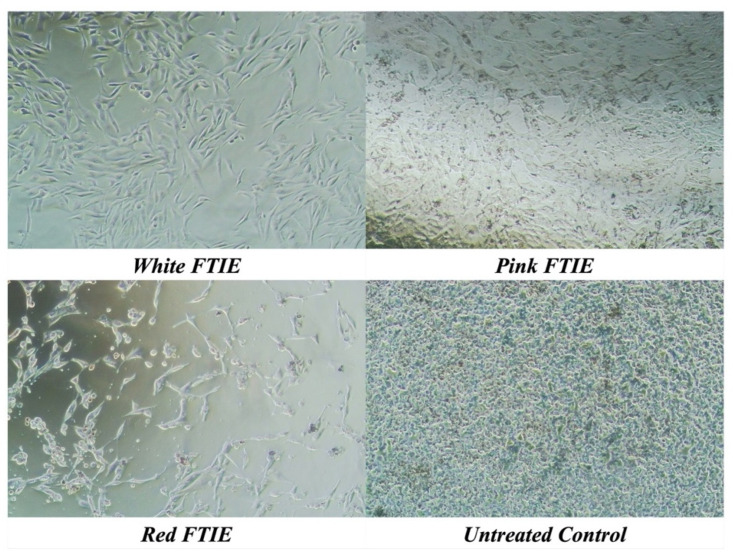
B16F10 cell density was observed under phase-contrast microscopy after exposure to FTIEs at a 2000 µg/mL concentration.

Cell–based cosmeceutical activity assays were conducted to evaluate the effects of FTIEs on various cellular processes. Before conducting the activity test, the toxicity of FTIEs against all tested cells was evaluated to determine the sample’s safety. The cell viability assay is essential because it can introduce artifacts into the interpretation of assay results [15]. The MTT assay measured the viability of cells exposed to FTIEs at concentrations ranging from 62.5 to 1000 µg/mL. The exposure time was by the cell-based activity testing protocol. L929 fibroblasts and RAW 264.7 murine monocyte/macrophages were employed after 24 h of exposure to FTIEs to test collagen synthesis and anti-inflammation. Therefore, an MTT assay was performed on L929 fibroblasts and RAW 264.7 murine monocyte/macrophages after 24 h of exposure to FTIEs. The B16F10 cells were used to test the effectiveness of a melanogenesis inhibitor. The experiment followed the method described by Kim et al. [16], where the cells were treated with the sample for 72 h before measuring the melanin content. Furthermore, the cell-free tyrosinase inhibitory activity of FTIEs revealed an IC_50_ of 3.5–4.4 mg/mL (Table 2). As a result, the cell viability of B16F10 was tested after 72 h of exposure to FTIE concentrations ranging from 78 to 5000 µg/mL to cover the IC_50_ of cell-free antityrosinase findings. Figure 3A–C demonstrate the viability of three types of cells: B16F10 murine melanoma cell, RAW264.7 mouse monocyte/macrophage, and L929 fibroblast. The normal cell lines, L929 fibroblast, and RAW 264.7 mouse monocyte/macrophage were unaffected by FTIEs at all concentrations tested (ranging from 62.5 to 1000 µg/mL). Their viability values remained close to 100 percent. However, the B16F10 mouse melanoma cell, a cancer cell, died after exposure to FTIEs. The pink FTIE was the least toxic to B16F10 cells, with a 50% lethal concentration (LC_50_) of 623.9 ± 54.4 µg/mL, in contrast to the white and red FTIEs, which had LC_50_ values of 329.5 ± 3.2 and 115.5 ± 4.8 µg/mL, respectively. Figure 4 displays the density of B16F10 mouse melanoma cells, as observed under a phase-contrast microscope, following exposure to FTIEs at the concentration of 2000 µg/mL. The results illustrate a lower cell density in the FTIEs-exposed group compared to the untreated control group. Krajarng et al. [3] revealed that flavonoids have been shown to have antioxidant properties, tumor cell growth inhibitory activity, and apoptosis induction in cancer cell lines. The toxicity levels of each variant of FTIE toward B16F10 cells had a positive correlation with their respective total flavonoid content. Notably, the red FTIE displayed the highest flavonoid content, corresponding to the highest observed cytotoxicity. Conversely, the pink FTIE exhibited the lowest flavonoid content, resulting in comparatively lower cell death. In addition, similar high toxicity against B16F10 cells of essential oil from *E. elatior* leaves [17] and a 50% hydroglycol extract of *E. elatior* flowers [3] while being less detrimental to normal fibroblast cell lines and normal Vero cells were reported. Krajarng et al. [3] suggested that *E. elatior* flower extract induced caspase-independent cell death via down-regulation of ERK and Akt pathways in B16 F10 cells; thus, this may be useful to further investigate as a chemopreventive or chemotherapeutic agent in the treatment of melanoma. However, their investigations did not describe the specific mechanism of cellular selectivity towards B16F10 [3,17].

Consequently, for this study, it can be realized that the FTIEs induced lethal effects on B16F10 melanoma cells at concentrations below the IC_50_ value of anti-tyrosinase activity, which were shown to be between 3000 and 4000 µg/mL (Table 2). Therefore, the assessment of cell-based melanogenesis is not feasible in this context.

#### 2.3.2. Fibroblast Collagen Synthesis Induced by FTIEs

**Figure 5:**
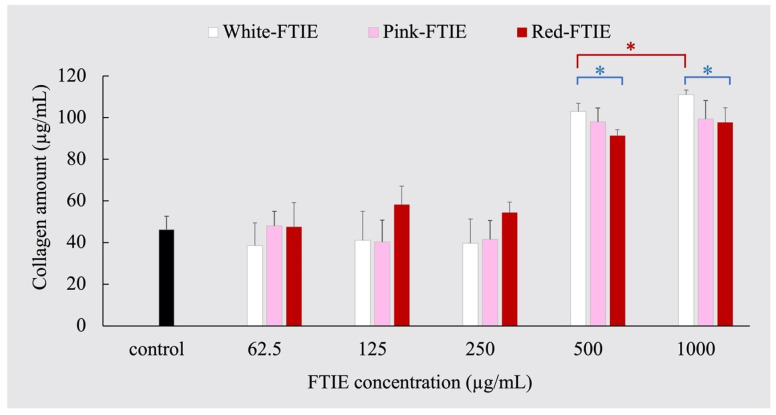
Collagen type I production in L929 fibroblast cell line when treated with various concentrations of FTIEs. Data expressed as a mean ± SD, *n* = 4. The symbol * denotes that the compared data differed significantly (*p*-value < 0.05).

In aging processes, the decrement of fibroblast collagen synthesis leads to wrinkle development. The ability of FTIEs to induce collagen synthesis in fibroblasts was investigated. The Sircol^TM^ soluble collagen test assessed collagen type I production in L929 fibroblast cells treated with FTIEs. The amount of collagen generated by fibroblast cells after exposure to various concentrations of the white, pink, and red-FTIE and the untreated control are displayed in Figure 5. The amount of collagen generated by fibroblasts exposed to FTIEs at concentrations lower than 250 µg/mL ranged from 40 to 50 µg/mL, which was not significantly different from the untreated control (*p*-value > 0.05). However, increasing the FTIE sample concentration to 500 µg/mL resulted in the fibroblast generating significantly greater amounts of collagen, 90–110 µg/mL, than the untreated control (*p*-value < 0.05).

There was a significant distinction in stimulating collagen production between each FTIE variant (*p*-value < 0.05), which did not correlate to the TPC, TFC, or cell-free activity results shown in Section 2.1 and Section 2.2. For cell-based collagen production activity, the white FTIE had the strongest effect and produced the highest collagen amount, while the red FTIE had the lowest effect. When the FTIE concentration increased from 500 µg/mL to 1000 µg/mL, only the white FTIE variety significantly increased collagen production (*p*-value < 0.05), while the red and the pink FTIE did not (*p*-value > 0.05).

Therefore, the red FTIE and the white FTIE are effective for both cell-free anti-collagenase activity (as demonstrated in Table 2, Section 2.2) and inductive collagen synthesis activity. These two activities have a synergistic effect on collagen production, which contributes to anti-aging, reduces wrinkles, and makes skin more resilient. In addition, the increased collagen synthesis is a positive indicator that FTIE may be able to accelerate the wound healing process, as will be discussed in the following section.

#### 2.3.3. Cell Migration Induced by FTIEs

**Figure 6:**
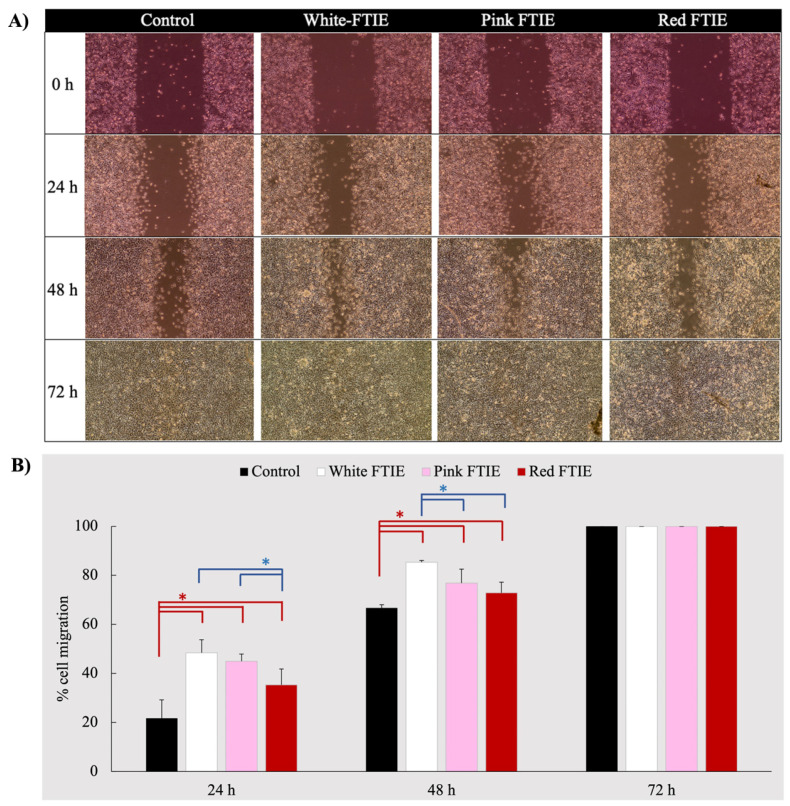
The wound was examined under an inverted light microscope (**A**) and a percentage of cell migration (**B**) after exposure to white, pink, and red-FTIEs for 24, 48, and 72 h. Data are expressed as a mean ± SD, *n* = 4. The symbol * denotes that the compared data differed significantly (*p*-value < 0.05).

The wound-healing process aims to restore the structure and function of the damaged tissue to its original state. The process initiates with a hemostatic response aimed at minimizing blood loss, followed by the activation of inflammatory cells, typically neutrophils and monocytes, which migrate to the site of damage to eradicate pathogens and remove tissue debris. The proliferative phase involves fibroblast and epithelial cell proliferation, the formation of new blood vessels, and synthesizing the extracellular matrix. Subsequently, the process transitions into the remodeling stage, which encompasses the closure of the wound, the synthesis of collagen, the reestablishment of epithelial tissue, and the formation of scar tissue [18]. Collagen is a key extracellular matrix component that plays multiple roles in healing [19]. According to the results of the experiment above (Section 2.3.2), FTIEs can stimulate fibroblast cells to produce considerable amounts of collagen, making them interesting to examine for enhancing and accelerating wound healing activity. In this section, the scratch assay was employed to investigate the effect of FTIEs on cell migration stimulation, which accelerated wound closure in relation to the untreated control. The concentration of FTIEs of 500 µg/mL, which corresponded to the greatest collagen synthesis stimulation effect obtained from all varieties of FTIE (Section 2.3.2), was chosen to evaluate the effect of FTIEs on cell migration. The wound examined under an inverted light microscope after 24, 48, and 72 h of FTIE exposure is illustrated in Figure 6A. The distance of the wound at each time point was estimated in comparison to the initial as a percentage of cell migration, as shown in Figure 6B.

The percentage of cell movement corresponded with the amount of collagen produced, with the white FTIE having the largest effect, followed by the pink and the red FTIE. However, after 24 and 48 h of exposure, all three FTIE varieties gave significantly greater cell migration rates than the control (*p*-value < 0.05). This demonstrated that all FTIE variants could accelerate wound healing processes compared to the untreated control.

In addition, the level of reactive oxygen species (ROS) substantially affects the natural processes of wound healing. When ROS levels are suitable, it is advantageous for accelerating wound healing. However, if the level of ROS is high enough to create oxidative stress, it impairs the healing process, causing cellular damage and chronic wounds [20]. Therefore, using substances with antioxidant activity to limit ROS amounts will also promote wound healing. Overall, FTIEs possess antioxidant activity, effectively induce fibroblast cells to produce collagen, and accelerate cell migration, all of which should contribute to the acceleration of the wound healing process. As a result, employing FTIEs in developing products for wound care is feasible. Even though FTIEs can stimulate fibroblasts to produce collagen, excessive collagen synthesis during the wound-healing process may increase the risk of keloid formation. Before adopting FTIEs as a wound treatment, it is recommended to further investigate the potential for FTIEs to promote keloid formation in wound healing models.

### 2.4. Anti-Inflammation Activity of FTIE

The inflammatory phase is the first and most important stage in the wound healing process. However, if the inflammatory phase becomes too intense and persists for a long time, it can result in an increased secretion of cytokines that promote inflammation, such as interleukin-1β (IL-1β), interleukin-6 (IL-6), and tumor necrosis factor-α (TNF-α). These cytokines can cause fibrosis and scarring formation, as well as hinder proper wound healing, which can slow down the progression to subsequent stages. The acceleration of the wound healing process has been demonstrated through the downregulation of pro-inflammatory cytokines. Therefore, the use of substances with anti-inflammatory activities that inhibit the production of pro-inflammatory cytokines, such as IL-1β, IL-6, and TNF-α, is a therapeutic target that can control the skin’s wound healing process [18]. 

**Figure 7:**
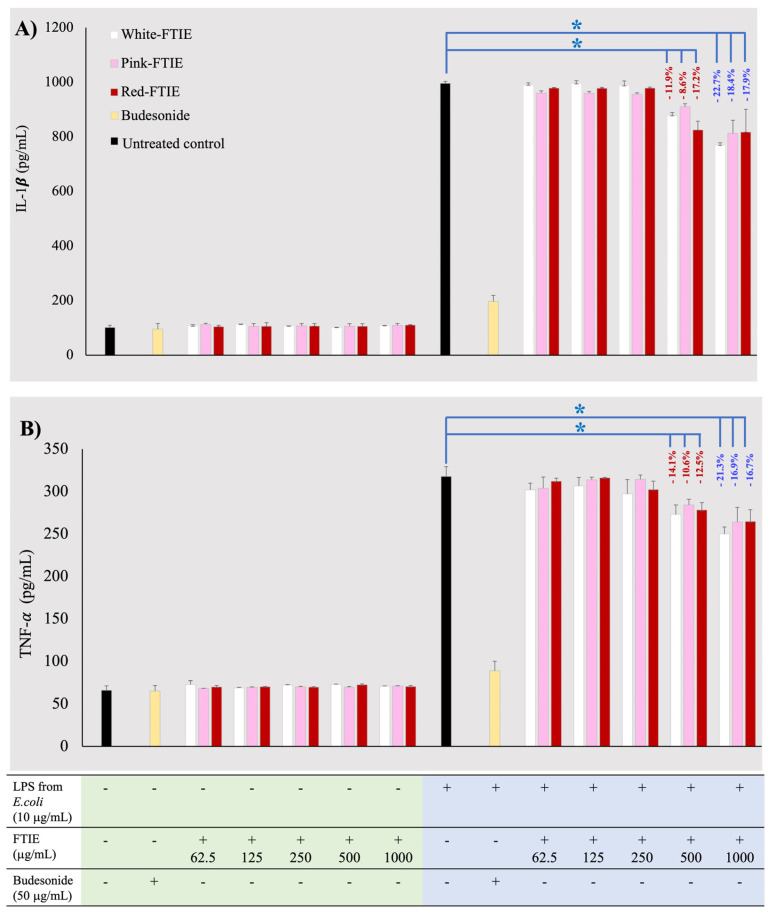
In vitro cytokine level results: (**A**) IL-1β, (**B**) TNF-α produced from RAW264.7 monocyte/macrophage cells or LPS from *E. coli* stimulated-mouse RAW264.7 monocyte/macrophage cells following exposure to white, pink, and red FTIEs, budesonide (positive control) and culture medium (negative control). Data expressed as a mean ± SD, *n* = 4. The symbol * denotes that the compared data differed significantly (*p*-value < 0.05).

FTIEs were first examined for their ability to elicit an immunological response, as shown in Figure 7A,B. The finding revealed that IL-1β and TNF-α, the studied pro-inflammatory cytokines produced from RAW 264.7 mouse monocyte/macrophage cells after exposure to various concentrations of FTIEs, were not significantly different from the untreated control. This demonstrated that FTIEs were safe and did not elicit an immunological response. This study used lipopolysaccharide (LPS) from gram-negative bacteria to stimulate cytokine production in RAW 264.7 murine monocyte/macrophage cells. Budesonide, a glucocorticosteroid, is an effective immunomodulator that lowers cytokine levels [21]. Budesonide (50 μg/mL) was used as a positive control in this study, and it was able to significantly inhibit the cytokine generated by LPS-stimulated RAW 264.7 monocytes, as shown in Figure 7A,B. FTIEs were investigated for their ability to suppress cytokine production in response to LPS; Figure 7A,B shows IL-1β and TNF-α levels, respectively. The results demonstrated that FTIEs, at concentrations greater than 500 µg/mL, could suppress the produced cytokine by 8.6–22.7%. However, cytokine levels decreased significantly compared to the untreated control group (*p*-value < 0.05). There were no statistically significant differences between FTIE variants in their capacity to suppress cytokine production (*p*-value > 0.05). This suggests that all variants of FTIE have a relatively weak anti-inflammatory effect.

## 3. Methods

### 3.1. Materials

*Etlingera elatior* (white, pink, and red inflorescence variety) were collected from the same garden in Pathumtani province, Thailand. The inflorescence was cut from the plant in the morning, at the mature stage with sizes of approximately 10 cm × 10 cm. 2,2-diphenyl-1-picrylhydrazyl (DPPH), 2,2′-azino-bis (3-ethylbenzothiazoline-6-sulfonic acid) (ABTS), 3,4-dihydroxy-L-phenylalanine (L-DOPA), 3-(4,5-dimethylthiazol-2-yl)-2,5-diphenyl-tetrazolium bromide (MTT), rutin, chlorogenic acid, gallic acid, potassium persulfate, kojic acid, epigallocatechin gallate (EGCG), mushroom tyrosinase enzyme, N-[3-(2-furyl)acryloyl]-Leu-Gly-Pro-Ala (FALGPA), collagenase enzyme from *Clostridium histolyticum*, Tricine buffer, were purchased from Sigma-Aldrich (St. Louis, MO, USA). Folin–Ciocalteu’s reagent was procured from Merck (Rahway, NJ, USA). Dulbecco’s Modified Eagle’s Medium (DMEM), fetal bovine serum (FBS), trypsin-EDTA, penicillin, and streptomycin were purchased from Gibco^®^ (Carlsbad, CA, USA). RCI Labscan (Bangkok, Thailand) purchased chromatographic solvent and other reagent-grade solvents.

### 3.2. Torch Ginger Inflorescent Preparation and Extraction

The bracts were removed and rinsed repeatedly to remove dirt, insects, and other foreign material. The cleaned bracts were cut into small pieces and dried in the hot air oven at 50 °C for 18 h. The dried flowers were afterward pulverized into a fine powder using an herbal blender (Spring Green Evolution, Bangkok, Thailand). Ethanol (50%) was utilized as a menstruum in a 1:6 ratio with fine inflorescence powder. The mixture was shaken at room temperature for 24 h in a shaking bath and filtered through Whatman No. 1 filter paper. The extraction procedure was repeated three times. The portions extracted from three replications were combined and concentrated using a rotary evaporator. The resulting concentrated extraction liquid was then freeze-dried using the following conditions: the sample was frozen at −80 °C, primary drying at −85 °C for 24 h, and secondary drying at 35 °C for 2 h. The freeze-dried torch ginger inflorescence extracts (FTIEs) were then collected (Figure 1).

### 3.3. Determination of the Secondary Metabolite Content of FTIEs

#### 3.3.1. Total Phenolic Content

The total phenolic content (TPC) of FTIE samples was determined using the method described by Saeed et al. [22] with some modifications. In brief, the reaction was conducted by combining 20 μL of FTIEs with 100 μL of 10% *v*/*v* Folin–Ciocalteu reagent and 80 μL of 7.5% *w*/*v* sodium carbonate (Na_2_CO_3_). The mixture was then incubated at room temperature in the dark for one hour. After that, the reaction mixture’s absorbance at 765 nm was determined. The gallic acid calibration curve determined and expressed the total phenolic content as milligrams of gallic acid equivalents per gram of FTIE powder (mg GAE/g).

#### 3.3.2. Total Flavonoid Content

The total flavonoid content of FTIE samples was determined following the method described by Saeed et al. [22]. The reaction contained 300 μL of FTIEs, 3.4 mL of 30% ethanol, 100 μL of 0.5 M sodium nitrite, and 150 μL of 0.3 M AlCl_3_·6H_2_O. The reaction mixture was incubated at room temperature for five minutes, and then 1 mL of 1 M NaOH was added and mixed thoroughly. The mixture absorbance was measured at 506 nm. The calibration curve of rutin was prepared in the concentration range of 0.1–1 mg/mL. The total flavonoid content was calculated and expressed as milligrams of rutin equivalents per g of FTIEs (mg RE/g).

#### 3.3.3. Total Caffeoylquinic Acid Content

The caffeoylquinic acid or CQA content of FTIEs was determined by the molybdate assay described by Chan et al. [7]. The molybdate reagent contained 16.5 g of sodium molybdate, 8.0 g of dipotassium hydrogen phosphate, and 7.9 g of potassium dihydrogen phosphate dissolved in 1000 mL of deionized water and pH adjusted to 6.5. To determine CQA content, 200 μL of the FTIE sample was mixed with 10 mL of the molybdate reagent and incubated for 10 min before the absorbance of the mixture was measured at 370 nm. The calibration curve of chlorogenic acid was prepared in the concentration range of 0.1–1 mg/mL. The CQA content was calculated and expressed as milligrams of chlorogenic acid equivalents per g (mg CQAE/g) of FTIE.

#### 3.3.4. Chlorogenic Acid and Rutin Content Determination by HPLC

The analytical method for simultaneous determination of chlorogenic acid and rutin in *E*. *elatior* extract was conducted using an HPLC instrument (Dionex^®^ Ultimate 3000 RS, Thermo Scientific, Waltham, MA, USA) comprising a quaternary pump, an autosampler, a column oven, and a diode array detector. A C18 HPLC column (4.6 mm × 150 mm, 5 microns; BDS Hypersil^®^, Thermo Scientific, Waltham, MA, USA) was utilized as a stationary phase. The mobile phase consisted of 0.1% *v*/*v* trifluoroacetic acid (A) and methanol (B). The gradient elution of the mobile phase was set at a flow rate of 1.0 mL/min with the following program: 0–5 min, 95%A: 5%B; 5–20 min, 95–75%A: 5–25%B; 20–30 min, 75–30% A: 25–70% B; 30–35 min, 30%A: 70%B; 35–40 min, 95%A: 5%B. Six concentrations of chlorogenic acid and rutin mixed standard solutions were prepared at 20–250 µg/mL and 10–100 µg/mL, respectively. The extract sample solutions were prepared with a 10 mg/mL methanol concentration. Before injection into the HPLC system, the solutions were filtered through a 0.45 µm membrane filter. Five microliters of the sample solutions were injected into the chromatographic system. The simultaneous detection of chlorogenic acid and rutin occurred at a wavelength of 257 nm. The extracts’ chlorogenic acid and rutin contents were calculated against the calibration curve.

### 3.4. Cell-Free Bioactivity of FTIEs

#### 3.4.1. Antioxidant Activity

##### 2,2-Diphenyl-1-picrylhydrazyl (DPPH) Radical Scavenging Activity

DPPH radical scavenging activity was determined using the method of Baluchamy and Subramanian [23] with some modifications. The FTIE sample was dissolved in 50% ethanol to produce a concentrated FTIE solution, which was then diluted to the desired concentration with purified water. In each well of a 96-well plate, 100 μL of FTIE samples at various concentrations (8–1000 µg/mL), and standard gallic acid (0.8–100 µg/mL), or purified water (control) was mixed with 100 μL of 0.25 mM DPPH in ethanol. The reaction mixture was incubated in the dark at room temperature for 30 min. Absorbance was measured at 517 nm using a microplate reader (Biohit^®^ 830, Biohit, Helsinki, Finland). Antioxidant activity was calculated by determining the percentage inhibition using Equation (1).

(1) $$\%\mathrm{inhibition}=\frac{\left[{\mathrm{Abs}}_{control}-{\mathrm{Abs}}_{sample\;or\;standard}\right]}{{\mathrm{Abs}}_{control}}\times 100$$

##### 2,2′-Azino-bis (3-Ethylbenzothiazoline-6-sulfonic Acid) (ABTS) Radical Scavenging Activity

The ABTS radical scavenging activity was assessed using the methodology described by Saeed et al. [11] with some modifications. The ABTS^●+^ reagent was made by combining a 7 mM ABTS solution with an equal volume of potassium persulfate solution (2.45 mM). The resulting mixture was subsequently incubated in the dark at room temperature for 12–16 h. Before adding the assay, the ABTS^●+^ reagent (2 mL) was diluted to 100 mL with distilled water, and the resulting solution should have given an absorbance at 734 nm in the range of 0.7 ± 0.05. The reaction was performed in individual wells of a 96-well plate to assay for ABTS radical scavenging activity. Each well contained 20 μL of the FTIE sample (8–1000 µg/mL) or standard gallic acid (0.8–100 µg/mL) at various concentrations or purified water (control), which was mixed with 180 μL of diluted ABTS^●+^ reagent solution. After 6 min of incubation, the absorbance was measured at a wavelength of 734 nm using a microplate reader (Biohit^®^ 830, Biohit, Helsinki, Finland). Antioxidant activity was determined by calculating the percentage inhibition following Equation (1).

#### 3.4.2. Anti-Tyrosinase Activity

The anti-tyrosinase activity of FTIEs was measured using the method described by Whangsomnuek et al. [10]. In brief, FTIE samples (0.08–10 mg/mL) or standard kojic acid (0.008–0.5 mg/mL) (20 μL) at various concentrations or phosphate-buffered solution pH 6.8 (control) were mixed with 40 μL of mushroom tyrosinase enzyme (200 unit/mL) and 140 μL of 20 mM phosphate-buffered solution pH 6.8. Following a 10 min incubation period, 40 μL of 2.5 mM of L-DOPA was introduced into each well of a 96-well plate. The plate was then incubated for 20 min at room temperature. The absorbance measurement was conducted at a wavelength of 470 nm for the reaction mixture, and the tyrosinase inhibition percentage was calculated using Equation (1).

#### 3.4.3. Anti-Collagenase Activity

The activity of the enzyme anti-collagenase was determined using a modified method published by Sooksomboon et al. [24]. A solvent consisting of Tricine buffer with a concentration of 50 mM and a pH of 7.5, supplemented with 10 mM CaCl_2_ and 400 mM NaCl, was used to prepare collagenase enzyme, collagen substrate (FALGPA), FTIE samples, and standard EGCG to the required concentration. To initiate the reaction, a pre-incubation step was performed by combining 120 μL of FTIE samples (8–1000 µg/mL) or standard EGCG (8–1000 µg/mL) with 15 μL of collagenase enzyme derived from *Clostridium histolyticum* (2.5 mg/mL). Following a 15 min incubation period at room temperature, 15 μL of 0.8 mM FALGPA was introduced into the reaction. Subsequently, the absorbance at a wavelength of 340 nm was monitored at 2 min intervals throughout 20 min. The change in absorbance (ΔAbs340 nm) was determined by subtracting the initial absorbance of FALGPA at 340 nm from the absorbance measured after a 20 min reaction period. The calculation of the anti-collagenase activity of the sample extracts was performed using Equation (2):

(2) $$\%\mathrm{inhibition}=\frac{{[\Delta \mathrm{Abs}340\;\mathrm{nm}}_{control}-{\Delta \mathrm{Abs}340\;\mathrm{nm}}_{sample\;or\;standard}]}{{\Delta \mathrm{Abs}340\;\mathrm{nm}}_{control}}\times 100$$

In Section 3.4, the FTIE concentration exerting 50% of inhibitory activity (IC_50_) in the unit of μg/mL was calculated using the corresponding equation in Table 3.

### 3.5. Cell-Based Bioactivity of FTIEs

#### 3.5.1. Cell Culture Condition

The mouse fibroblast cell line L929 (Chinese Academy of Preventive Medical Sciences, Beijing, China), the RAW 264.7 mouse monocyte/macrophage cell line (ATCC TIB-71, Manassas, VA, USA) or the B16F10 murine melanoma cell (ATCC CRL-6475™, Manassas, VA, USA) was cultured in Dulbecco Modified Eagle Medium (DMEM) containing 10% fetal bovine serum (FBS), antibiotics (100 U penicillin, and 100 U streptomycin per mL) under 5% CO_2_ at 37 °C. The medium was changed every two days. When cells reached confluence, they were harvested using 0.25% trypsin-EDTA (Gibco^®^, Carlsbad, CA, USA), followed by adding fresh culture medium to create a new single-cell suspension for further incubation.

#### 3.5.2. Cell Viability Assay

A quantity of 100 μL of cells at a concentration of 1 × 10^5^ cells/mL was seeded in a 96-well plate with a complete medium and incubated for 24 h. FTIEs were diluted with culture medium before being filtered through a 0.2 µm sterile syringe filter (Corning^®^, Kaiserslautern, Germany). L929 and RAW 264.7 cells were exposed to the samples (62.5–1000 μg/mL) for 24 h by the tested methods for collagen synthesis (Section 3.5.3) and anti-inflammation (Section 3.5.5), respectively, while the cell viability assay of B16F10 was assessed following exposure to FTIEs (78.1–5000 μg/mL) for 72 h per the procedure for a melanogenesis inhibitory experiment reported by Kim et al. [16]. Cell viability was assessed using a 3-(4,5-dimethylthiazol-2-yl)-2,5-diphenyl-tetrazolium bromide (MTT) solution with a 5 mg/mL concentration. To perform the test, 80 µL of fresh media and 20 µL of the MTT solution were added to the cells, which were then incubated at 37 °C under 5% CO_2_ for 4 h. After that, the MTT media were removed, and 100 µL of 100% dimethyl sulfoxide was added to dissolve the formed formazan salt. Absorbance was recorded using a microplate reader (Biohit^®^ 830, Biohit^®^, Helsinki, Finland) at 570 nm. The percentage of cell viability was calculated compared to the untreated control using Equation (3):

(3) $$\%\mathrm{Cell}\;\mathrm{viability}=\frac{{\mathrm{Abs}\;570\;\mathrm{nm}}_{FTIEs\;treated\;cell}}{{\mathrm{Abs}\;570\;\mathrm{nm}}_{untreated\;control}}\times 100$$

#### 3.5.3. Determination of Soluble Collagen Production Induced by FTIEs

L929 murine fibroblast cells were cultivated and processed like the cytotoxicity test described in Section 3.5.2. Based on the findings of Section 3.5.2, FTIEs were added to each well in a safe concentration range (62.5–1000 µg/mL). After 24 h of incubation in a CO_2_ incubator, cell supernatants were collected, and the amount of generated soluble collagen was evaluated using the Sircol^™^ collagen assay kit (Biocolor^®^ Ltd., Carrickfergus, UK). A microplate reader (Biohit^®^ 830, Biohit, Helsinki, Finland) was used to measure the absorbance of the reaction at a wavelength of 500 nm. All experiments were carried out in four replications. Based on a standard curve of standard soluble collagen type 1 provided in the assay kit, the measured absorbance was converted to the amount of generated collagen. 

#### 3.5.4. In Vitro Wound Healing Induced by FTIEs

The scratch wound healing test was employed to assess the migration process of fibroblast cells after stimulation with FTIEs. The method employed in this research followed the approach outlined by Balekar et al. [25]. In this study, L929 fibroblast cells were seeded at a density of 5 × 10^4^ cells per well of a 6-well plate. The cells were then incubated until a confluent monolayer was formed. A sterile pipette tip generated a linear scratch on the monolayer. Cellular debris was removed and substituted with 2 mL of Dulbecco’s Modified Eagle Medium (DMEM) containing a sample of FTIEs at a 500 μg/mL concentration. The untreated group was used as a control. The images were observed under a microscope at a magnification of 10× and were captured at four-time points: initial, 24, 48, and 72 h. The scratch closure distance of the images was quantitatively evaluated using computational software (ImageJ1.42q/Java1.6.0 10), and the percentage of cell migration rate was estimated. The mean of four independent replicates was calculated.

#### 3.5.5. Anti-Inflammation: FTIEs Efficacy to Suppress LPS-Induced Inflammatory Cytokine Production

The RAW 264.7 murine monocyte/macrophage cell line was employed in this investigation to produce inflammation cytokines, interleukin 1β (IL-1β), and tumor necrosis factor-α (TNF-α), which were stimulated by lipopolysaccharide (LPS) from *Escherichia coli*. The anti-inflammatory study method employed was adapted from the method outlined in a previous publication by Changsan et al. [26]. The safe concentration range of FTIEs on RAW 264.7 cells was determined using MTT tests, as outlined in Section 3.5.2. This information was then utilized to assess the impact of FTIEs on the suppression of cytokine production. A RAW 264.7 cell (10^4^ cells) was placed in each well of a 96-well plate. The effectiveness of FTIEs at various concentrations in reducing the inflammatory cytokine generated in the RAW 264.7 response to LPS from *E. coli* (1 µg/mL) stimulation was evaluated. The positive and negative controls were budesonide (50 µg/mL) and cell culture medium, respectively. The concentrations of the inflammatory cytokines IL-1β and TNF-α in the cellular supernatant were measured using the enzyme-linked immunoassay (ELISA) method. The Quantikine^®^ Colorimetric Sandwich ELISA test kit (R&D Systems, Minneapolis, MN, USA) was employed to quantify the concentrations of mouse IL-1β and mouse TNF-α. The experimental procedures were carried out per the directions outlined in the guidebook included with the kit.

#### 3.5.6. Statistical Analysis

All results were presented as mean and standard deviations. One-way analysis of variance (ANOVA) and student’s *t*-test were used to assess significant variations in the mean parameters at the significance level set at 0.05. Microsoft Excel for Mac Version 16.77 (Redmond, WA, USA) was used for all statistical analysis. 

## 4. Conclusions

FTIE extracted from red, pink, and white inflorescence variants contains significant phenolic, flavonoid, caffeoylquinic, chlorogenic, and rutin, thus proving to have excellent free radical scavenging activity. The tyrosinase enzyme, which is essential for the production of melanin, can be inhibited by all FTIE types nevertheless when used in extremely high concentrations. The red and the white FTIE effectively inhibited the collagenase enzyme responsible for collagen degradation. Based on the chemical composition and cell-free bioactivity, the red FTIE showed the most favorable outcomes, followed by the white and pink variants. In cell-based assays, FTIEs were toxic to murine melanoma cells while safe for normal fibroblast and monocyte cells. The white FTIE variant was the most effective at promoting fibroblasts to produce more collagen than the control, followed by the pink FTIE and the red FTIE, which were the least effective. These results correspond to their wound-healing efficacy. All FTIE variants possess a relatively weak anti-inflammatory effect. Among the three variants, the red FTIE exhibited the most optimistic potential as an anti-aging cosmeceutical component due to its free radical scavenging activity, anti-collagenase, and inductive collagen synthesis properties. For the use of FTIEs in wound care products, white FTIE could be advantageous due to its superior wound healing acceleration and collagen synthesis induction properties.

## Figures and Tables

**Table 1:** Total phenolic, flavonoid, caffeoylquinic acid, chlorogenic and rutin content of FTIEs (*n* = 3).

	TPC mg GAE/g FTIE	TFC mg RE/g FTIE	TCQAC mg CGA/g FTIE	CGA Content mg/g FTIE	Rutin Content mg/g FTIE
White-FTIE	120.59 ± 10.66	56.21 ± 1.93	22.00 ± 1.68	10.14 ± 0.19	1.07 ± 0.02
Pink-FTIE	102.83 ± 4.66	29.63 ± 3.40	16.04 ± 4.88	4.65 ± 0.08	1.89 ± 0.02
Red-FTIE	165.09 ± 8.60	96.70 ± 4.25	38.55 ± 2.72	12.78 ± 0.24	1.25 ± 0.02

TPC: Total phenolic content, TFC: Total flavonoid content, TCQAC: Total caffeoylquinic acid content, CGA: chlorogenic acid.

**Table 2:** Cell-free antioxidant, anti-tyrosinase, and anti-collagenase enzyme activities of FTIEs.

	IC_50_ (μg/mL)			
	Antioxidant Activity		Anti-Tyrosinase Activity	Anti-Collagenase Activity
	DPPH	ABTS		
White-FTIE	80.09 ± 9.70	309.83 ± 10.27	3500.33 ± 58.01	378.18 ± 65.90
Pink-FTIE	105.73 ± 5.02	495.93 ± 5.92	4427.00 ± 131.81	>1000
Red-FTIE	61.32 ± 4.50	239.67 ± 5.78	3514.33 ± 139.20	255.68 ± 19.06
Rutin	31.65 ± 0.03	570.17 ± 24.57	>1000	>1000
CGA	11.14 ± 4.86	256.50 ± 5.11	>1000	842.81 ± 11.13
Gallic acid	2.91 ± 0.54	14.67 ± 0.66	-	-
Kojic acid	-	-	53.60 ± 11.28	-
EGCG	-	-	-	385.40 ± 25.54

Data are mean ± SD from 4 replications of experiment.

**Table 3:** The equation employed to estimate the IC_50_ value for each activity in Section 3.4.

	Antioxidant Activity		Anti-Tyrosinase Activity	Anti-Collagenase Activity
	DPPH	ABTS		
White-FTIE	y = 0.2955x + 25.096 R^2^ = 0.9049	y = 0.1739x + 7.6862 R^2^ = 0.9165	y = 0.01x + 7.6803 R^2^ = 0.9627	y = 0.083x + 21.449 R^2^ = 0.9039
Pink-FTIE	y = 0.2844x + 19.434 R^2^ = 0.9103	y = 0.0860x + 6.9909 R^2^ = 0.9193	y = 0.0096x + 5.0238 R^2^ = 0.9881	ND
Red-FTIE	y = 0.5075x + 19.312 R^2^ = 0.9238	y = 0.1488x + 3.9321 R^2^ = 0.9809	y = 0.0117x + 9.065 R^2^ = 0.9399	y = 0.1946x + 4.2246 R^2^ = 0.9775
Rutin	y = 0.7615x + 24.602 R^2^ = 0.9288	y = 0.0646x + 12.819 R^2^ = 0.9173	>1000	ND
CGA	y = 1.7474x + 25.358 R^2^ = 0.9352	y = 0.1197x + 17.947 R^2^ = 0.9694	ND	y = 0.0695x − 5.0736 R^2^ = 0.9697
Gallic acid	y = 9.2615x + 27.149 R^2^ = 0.9441	y = 4.8410x − 9.4989 R^2^ = 0.9774	-	-
Kojic acid	ND	ND	y = 1.0279x − 2.3998 R^2^ = 0.9995	-
EGCG	-	-	-	y = 0.0701x + 18.529 R^2^ = 0.9710

## Data Availability

The data presented in this study are contained within the article.
